# YiQiFuMai Powder Injection Attenuates Coronary Artery Ligation-Induced Heart Failure Through Improving Mitochondrial Function via Regulating ROS Generation and CaMKII Signaling Pathways

**DOI:** 10.3389/fphar.2019.00381

**Published:** 2019-04-10

**Authors:** Yu Zhang, Ling Zhang, Yan Zhang, Xiaoxue Fan, Weiwei Yang, Boyang Yu, Junping Kou, Fang Li

**Affiliations:** ^1^State Key Laboratory of Natural Products, Jiangsu Key Laboratory of TCM Evaluation and Translational Research, Department of Complex Prescription of TCM, School of Traditional Chinese Pharmacy, China Pharmaceutical University, Nanjing, China; ^2^Nanjing Chinese Medicine Hospital Affiliated to Nanjing University of Chinese Medicine, Nanjing University of Chinese Medicine, Nanjing, China

**Keywords:** YiQiFuMai powder injection, heart failure, mitochondrial function, ROS generation, calmodulin dependent protein kinase II

## Abstract

The YiQiFuMai powder injection (YQFM), a traditional Chinese medicine (TCM) prescription re-developed based on Sheng-Mai-San, is widely applied for the treatment of cardiovascular diseases. However, its potential molecular mechanism remains obscure. The present study was designed to observe the effects of YQFM and underlying mechanisms on coronary artery ligation (CAL)-induced heart failure (HF) and cell hypoxia of 24 h oxygen-glucose deprivation (OGD) in neonatal rat ventricular myocytes (NRVMs). HF was induced by permanent CAL for 2 weeks in ICR mice. The results demonstrated that YQFM significantly attenuated CAL-induced HF via improving the cardiac function, cardiac systolic function, cardiac structure impairment, cardiac histological features and fibrosis. YQFM markedly attenuated mitochondrial dysfunction through improving mitochondrial morphology, increasing mitochondria membrane potential (Δψm), mitochondrial ROS generation and expression of Mitofusin-2 (Mfn2), meanwhile, decreasing phosphorylation of dynamin-related protein 1 (p-Drp1). Mechanistically, YQFM could significantly decrease the expression of isoforms of nicotinamide adenine dinucleotide phosphate (NADPH) oxidase subunit NADPH oxidase 2 (NOX2), p67^phox^ and NADPH oxidase 4 (NOX4), ultimately reducing reactive oxygen species (ROS) generation. In addition, YQFM could down-regulate expression of calcium voltage-gated channel subunit α1C (CACNA1C) and phosphorylation of calmodulin dependent protein kinase II (p-CaMKII). These results suggest that YQFM ameliorates mitochondrial function in HF mice, partially through inhibiting ROS generation and CaMKII signaling pathways. Therefore, the present study provided scientific evidence for the underlying mechanism of YQFM.

## Introduction

Heart failure (HF) is an intractable disease and associated with substantial economic costs in the world (Jessup et al., 2009; Corrao and Maggioni, 2014). Ischemic heart disease (IHD), also known as coronary artery disease (CAD), is the cause of 52% of incident HF in the general population under 75 years (Fox et al., 2001). Currently, most patients with a recent or remote history of myocardial infarction (MI) or HF commonly treat with diuretics, neurohormonal antagonists, β-blockers, and ACE inhibitors (Voora and Ginsburg, 2012). These treatments although beneficial in promoting some symptom relief and temporarily impeding disease progression, often do not fully address the underlying causes of HF (Benjamin et al., 2017).

Mechanisms underlying the development of HF are multiple and complex, including calcium ion (Ca^2+^) overload, mitochondrial dysfunction, ventricular remodeling, apoptosis, and so on. Mitochondrial dysfunction is an important event in the development of HF (Bayeva et al., 2013). Mitochondria are vital for cardiac function, as they support continuous cycles of contraction and relaxation through providing energy necessary. Moreover, mitochondria participate in the synthesis of cellular components, calcium buffering, and triggering of cell death signals. Almost certainly, mitochondrial dysfunction has been related to several cardiovascular disorders, such as hypertension, cardiac hypertrophy, ischemia/reperfusion (I/R) and HF (Archer, 2013; Ong et al., 2013). Besides, the possible causes leading to mitochondrial dysfunction include oxidative stress, Ca^2+^ disorder, reduction of mitochondrial biosynthesis and so on, all of which are also closely linked to the development of HF.

With more and more clinic demand of drugs for the treatment of HF, many researchers have taken great notice of traditional Chinese medicine (TCM), because of their distinct advantage in holistic adjustment. The YiQiFuMai powder injection (YQFM) with better efficacy and fewer side effects compared with standard medical treatments, has been re-developed from a well-known TCM complex prescription Sheng Mai San (Xu, 2009), which is composed of three herbs: Radix of *Panax ginseng* C.A. Mey. (Araliaceae), Radix of *Ophiopogon japonicus* (Thunb.) Ker Gawl (Liliaceae), and Fructus of *Schisandra chinensis* (Turcz.) Baill (Schisandraceae). YQFM was provided by Tasly Pharmaceutical Co., Ltd. (Tianjin, China). Moreover, YQFM is a clinically listed drug with stable and reliable quality. Twenty-one compounds in YQFM have been identified using UFLC-IT-TOF/MS, and the concentration ranges in the ten batches of YQFM were 0.31–0.37 mg/g for the total three ophiopogonins, 6.66–9.24 mg/g for the total fifteen ginsenosides, and 0.13–0.17 mg/g for the total three lignans. These results indicated that the difference among these ten batches of YQFM is small (Liu et al., 2016). Besides, the determination of Ginsenoside Rg1, Ginsenoside Rb1 and Schisandrin in YQFM by the HPLC-DAD-ELSD eluted system, their concentration is 0.3363 ± 0.0108 mg/g, 1.5061 ± 0.0498 mg/g and 0.09047 ± 0.0009 mg/g, respectively (Li et al., 2015). Previous researches have demonstrated that YQFM and its effective constituents could improve HF through inhibiting NF-κB activation and cytokines (Xing et al., 2013), as well as ameliorate hypoxia-induced myocardial injury (Feng et al., 2016). Moreover, YQFM could modulate MAPKs signaling pathway to attenuate coronary artery ligation (CAL)-induced myocardial remodeling and HF (Pang et al., 2017). Nevertheless, the potential mechanisms of YQFM against HF remain to be further illuminated.

In present study, YQFM was tested in a CAL-induced HF mouse model, and in neonatal rat cardiac myocytes (NRVMs) subjected to oxygen glucose deprivation (OGD), to further explore the possible underlying mechanisms of YQFM against HF.

## Materials and Methods

### Reagents and Drugs

YiQiFuMai powder injection was provided by Tasly Pharmaceutical Co., Ltd., (Tianjin, China, batch number 20161016). The proportions of Panax ginseng: Ophiopogon japonicas: Schisandra chinensis are 1: 3: 1.5. The YQFM is composed of the ethanol extract (78°C) of above three herbals. The yield of extracts was 23.64%. After extraction, other procedures include multiple filtration, lyophilization, and aseptic packaging (Pang et al., 2017). Apocynin (APO) (cat. no. S2425) and nifedipine (cat. no. S1808) were obtained from Selleck Chemicals (Houston, TX, United States). KN-93 (cat. no. A3532) was purchased from ApexBio (Houston, TX, United States). Captopril (cat. no. C4042-5G), Mdivi-1 (cat. no. M0199) were purchased from Sigma (St. Louis, MO, United States). Mito SOX Red FM (cat. no. M36008), Mito Tracker^®^Deep Red FM (cat. no. M22426) and Dulbecco’s modified Eagle medium (DMEM) were obtained from GIBCO/BRL (Life Technologies, Carlsbad, CA, United States). Fetal bovine serum (FBS) was from ScienCell (Carlsbad, CA, United States). 3-[4,5-Dimethylthiazol-2-yl]-2,5-diphenyl-tetrazolium bromide (MTT) was from AMRESCO (Cleveland, OH, United States). Dihydroethidium (DHE) (cat. no. S0063) and probe JC-1 (cat. no. C2006) were obtained from Beyotime Institute of Biotechnology (Shanghai, China). RIPA lysis buffer, protease inhibitor and enhanced chemiluminescence (ECL) reagent were from Vazyme Biotech (Nanjing, China). Antibody against GAPDH (cat. no. MB001), β-actin (cat. no. AP0060), CACNA1C (cat. no. BS60266) and Mfn2 (cat. no. BS71425) were from Bioworld Technology (St. Louis Park, MN, United States). Antibody against p-Drp1 (Ser616) (cat. no. 3455S) were obtained from Cell Signaling Technology (Boston, MA, United States). Antibody against Drp1(cat. no. ab56788), CaMKII (cat. no. ab134041), p-CaMKII (Thr286) (cat. no. ab171095), NOX2/gp91^phox^ (cat. no. ab129068), p67^phox^ (cat. no. ab109366) and NOX4 (cat. no. ab133303) were purchased from Abcam Technology (Cambridge, MA, United States).

### HPLC-MS Analysis

The preparation of YQFM injection and the method of HPLC-MS analysis were conducted as reported previously (Li et al., 2015). Triple-quadrupole tandem mass spectrometric detection was carried out on a Micromass Quattro micro^TM^ API mass spectrometer (Waters Corp, Milford, CT, United States) with an electrospray ionization (ESI) interface. The mobile phase consisted of acetonitrile (A) and water-0.1% acetic acid (B), and the gradient elution conditions were: 0–30 min, 8–18% A; 30–80 min, 18–50% A; 80–115 min, 50–100% A and then returned to the initial condition. The flow rate was 1.0 mL/min. Chromatographic separation was carried out at 30°C on an Alltima C18 column (4.6 mm × 250 mm, I.D., 5 μm, Serial No. 213040116, GRACE-Alltech, United States).

### Animals

ICR male mice (8-week-old) were obtained from the Experimental Animal Center of Yangzhou University (Yangzhou, China). They were housed individually in cages under hygienic conditions and placed in a constant temperature (23 ± 1°C), humidity (30–40%) and maintained on a 12 h light/dark cycle room. This study was carried out in accordance with the recommendations of “Guide for the Care and Use of Laboratory Animals, National Institutes of Health.” The protocol was approved by the “China Pharmaceutical University.”

### Surgical Preparation

The mice were anesthetized with chloral hydrate (4% chloral hydrate, ip). The HF model was induced by CAL as previously reported (Gao et al., 2010). Successful ligation of the coronary artery was confirmed by the occurrence of ST-segment elevation in electrocardiogram. Sham operated mice were performed the same process except left CAL. After ligation, the heart was rapidly put back into the intrathoracic space. After surgery, the surviving mouse were divided randomly into seven group: the Sham group, the Model group, the YQFM group (three doses of 0.13, 0.26, and 0.53 g/kg, ip), Captopril (0.16 g/kg, ip), the Sham administrated YQFM (0.53 g/kg, ip) (All dissolved in 0.9% sodium chloride). Both the Sham and the Model mice were administrated physiologic saline with an equal volume via intraperitoneal injection. 15 mice in each group were treated for 2 weeks (once a day).

### Echocardiographic Measurement

After 14 days treatment, according to previous study (Li et al., 2016), echocardiography was performed using the Vevo2100 imaging system with a 30-MHz probe. The following parameters were detected: left ventricular ejection fraction (LVEF) = (LVEDV-LVESV) / LVEDV, left ventricular endocardial fractional shortening (LVFS) = (LVD;d –LVD;s) / LVD;d. Based on these measurements of interventricular septum in diastole (IVS;d), left ventricle interior diameter in diastole (LVID;d) and left ventricle posterior wall in diastole (LVPW;d), the following parameters were calculated: left ventricular Mass (corrected), left ventricular volume in diastole (LV Vold;d) and relative wall thickness (RWT) = (IVS+LVPW) / LVID.

### Heart Hematoxylin-Eosin and Masson’s Trichrome Staining

After mice were sacrificed, the heart samples were separated and fixed in 10% phosphate-buffered formalin for 24 h, subsequently embedded in paraffin, sliced (4–5 μm). Histologic sections of tissues were stained with hematoxylin-eosin and Masson’s trichrome. The sections were pictured under a light microscope (DX45, Olympus Microsystems Ltd., Japanese).

### Transmission Electron Microscopy

Left ventricular samples peri against infarction were fixed with a 4% paraformaldehyde solution including 2.5% glutaraldehyde for 24 h and cut into 1 mm^3^ pieces. Then the sample was prepared according to the previous report (Lavorato et al., 2017), and the ultrastructure was detected using transmission electron microscopy (JEM-1001, JEOL Ltd., Tokyo, Japan). The mean number of mitochondria and ratio of major and minor axes of mitochondria were calculated.

### Primary Cultures and Identification of NRVMs

Neonatal rat ventricular myocytes were derived from 3 to 4 days old Sprague-Dawley rat according to previous report (Maass and Buvoli, 2007). Briefly, ventricular myocytes were enzymatically dissociated and enriched for cardiomyocytes. We routinely obtained contractile myocardial cell cultures with 98–99% myocytes, as evidences by microscopic observation of cell beating, and marked with cardiac troponin T in immunofluorescence. Images were obtained using a confocal scanning microscope (LSM700, Zeiss, Jena, Germany). NRVMs were cultured in DMEM with 10% FBS at 37°C in a humidified atmosphere of 5% CO_2_.

### OGD Injury in NRVMs

The OGD technique was applied based on a previously described protocol (Li et al., 2016) to mimic the ischemic injury *in vitro*. In our study, the OGD injury was produced by incubating with none glucose DMEM and exposed to a hypoxic environment of 94% N_2_, 5% CO_2_ and 1% O_2_ for 24 h at 37°C in a humidified N_2_/CO_2_ incubator. Cells were treated with YQFM (25–800 μg/mL), Captopril (Cap, 21.7 μg/mL), APO (200 nM), Nifedipine (10 μM) and KN-93 (10 μM) during the hypoxia.

### JC-1 Staining for Mitochondrial Membrane Potential in NRVMs

The cells were incubated with JC-1 staining solution (10 μg/mL) for 20 min at 37°C after each treatment. The cells were then washed twice with PBS. Finally, cells were immediately analyzed using a confocal scanning microscope (LSM700, Zeiss, Jena, Germany). Data were expressed as the ratio of red to green fluorescence intensity, which indicates the level of depolarization of the mitochondrial membrane (JC-1 aggregates: excitation/emission = 543/590 nm; JC-1 monomers: excitation/emission = 488/525 nm).

### Cell Viability

Cell viability was determined using MTT assay. Cells were seeded at a density of 15 × 10^4^ cells/well in 96-well plates. After treatments, MTT assay was performed as previous report [17]. Cells were incubated with MTT at a final concentration of 0.5 mg/mL for another 4 h. Then, the medium was discarded, 150 μL of DMSO was added with shaking for 10 min to dissolve the formazan crystals. The absorbance was read measured using a microplate reader (Epoch, BioTek, Norcross, GA, United States) at a detection wavelength of 570 nm, with a reference wavelength of 650 nm, and cell viability was expressed as a percentage of the untreated control values.

### Mitochondrial Morphology Analysis in NRVMs

For mitochondrial morphology assay, NRVMS were washed with PBS, and then incubated with 400 nM Mito Tracker^®^Deep Red FM (Molecular Probes) for 30 min at 37°C. Then the cells were washed with PBS. The structures of mitochondria were viewed by confocal microscopy (LSM700, Zeiss, Jena, Germany). (Mito Tracker^®^Deep Red FM: excitation/emission = 644/665 nm).

### Measurement of Mitochondrial ROS in NRVMs

For mitochondrial ROS assay, NRVMs were washed with PBS, and then incubated with 5 μM Mito SOX Red FM (Molecular Probes) for 10 min at 37°C. After washed with PBS, the mitochondrial ROS was viewed by confocal microscopy (LSM700, Zeiss, Jena, Germany). (Mito SOX Red FM: excitation/emission = 510/580 nm).

### Measurement of ROS

For intracellular ROS production, tissue section and cells were washed twice with PBS, and then incubated with ROS specific fluorescent probe dye DHE (5 μM) for 30 min at 37°C. After washing, the DHE fluorescence was observed with a confocal laser scanning microscope (LSM700, Zeiss, Jena, Germany). (Ethidium: excitation/emission = 488/525 nm).

### Western Blot Analysis

Left ventricular samples peri against infarction and cell samples were prepared in lysis buffer containing 1 mM PMSF. Equal amounts of protein samples were separated by denaturing SDS–PAGE, followed by transferring to polyvinyl difluoride (PVDF) membranes (Millipore Corporation, Bedford, MA, United States). The membranes were blocked with 3% BSA and stained with appropriate primary antibodies against GAPDH, β-Actin, Drp1, p-Drp1, Mfn2, CACNA1C, CaMKII, p-CaMKII, NOX2, p67^phox^ and NOX4 (dilution 1:8000, 1:1000, 1:1000, 1:500, 1:1000, 1:800, 1:2000, 1:2000, 1:5000, 1:5000, 1:2000, respectively) for 16 h at 4°C. After washing, membranes were probed with the HRP-conjugated secondary antibodies (dilution 1:8000, Bioworld, Louis Park, MN, United States). The antigen-antibody complexes were detected using enhanced chemiluminescence (ECL, Vazyme Biotech, Nanjing, China) and visualized by ChemiDoc^TM^ MP System (Bio-Rad). The results were quantified using Image Lab^TM^ software (version 4.1, Bio-Rad, Hercules, CA, United States).

### Statistical Analysis

Statistical analysis of data was performed using one-way analysis of variance (ANOVA) followed by Dunnett’s *post hoc* test for multiple comparisons using GraphPad Prism 6.0 (La Jolla, CA, United States). All data in the text and figures were expressed as mean ± SD. *P* < 0.05 was considered significant.

## Results

### HPLC-MS Analysis

**FIGURE 1:**
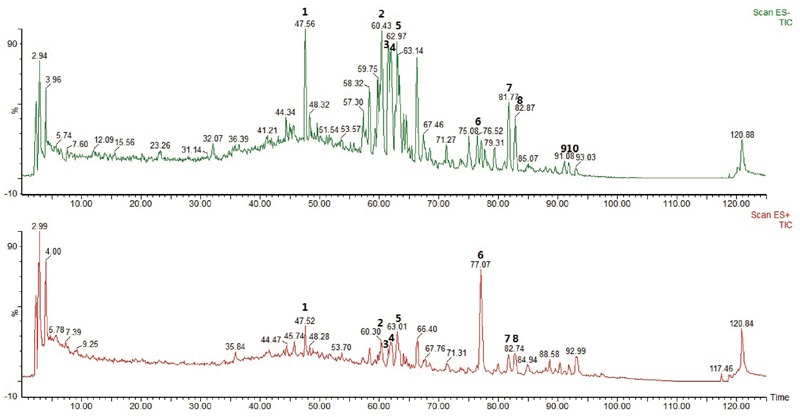
The base peak chromatograms of YQFM were analyzed by HPLC-MS in negative and positive ion mode. Identification of main compounds in YQFM as following: 1. Ginsenoside Rg1; 2. Ginsenoside Rb1; 3. Ginsenoside Rc; 4. Ginsenoside Rb2; 5. Ginsenoside Rd; 6. Schizandrol A; 7. 20(S)-ginsenoside Rg3; 8. 20(R)-ginsenoside Rg3; 9. Ginsenoside Rk1; 10. Ginsenoside Rg5.

The main components of YQFM were analyzed using HPLC-MS method. Representative MS base peak chromatograms of YQFM were shown (Figure 1). In present study, compounds were identified via comparison with previous literatures which were characterized based on their retention times and MS spectra (Li et al., 2015).

### YQFM Improved the Mitochondrial Function in CAL-Induced HF Mice

**FIGURE 2:**
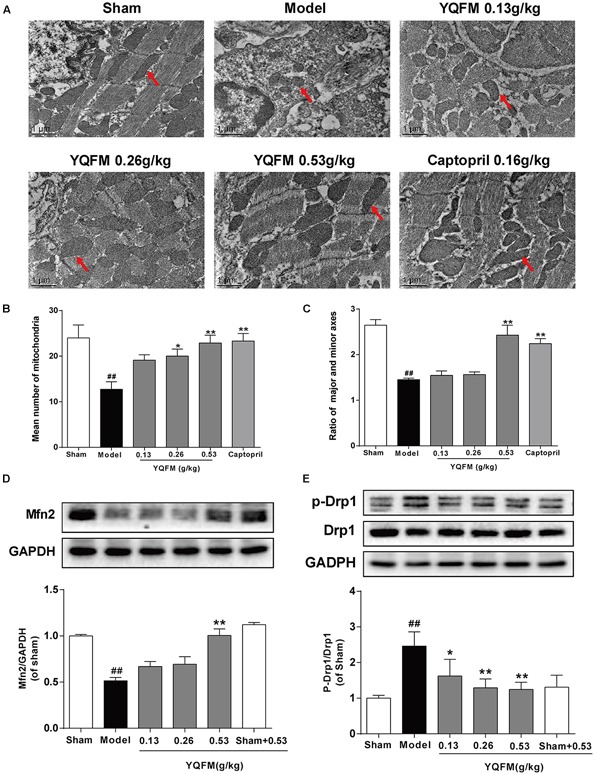
YQFM improved the mitochondrial function in CAL-induced HF mice. **(A)** Representative photomicrographs (× 13000) of the mitochondrial morphology. The red arrows represent the mitochondria. **(B)** The mean number of mitochondria was measured. **(C)** The ratio of major and minor axes of mitochondria was calculated. Expression of Mfn2 **(D)** and p-Drp1 **(E)** were determined by Western blot analysis. Results were presented as mean ± SD. ^##^*P* < 0.01 vs. Sham group, ^∗^*P* < 0.05, ^∗∗^*P* < 0.01 vs. Model group. *n* = 3.

Previous study has indicated YQFM had a good effect on CAL-induced HF (Pang et al., 2017). In present study, the results further demonstrated that YQFM could also ameliorate cardiac systolic function, cardiac structure impairment, cardiac histological features and fibrosis in CAL-induced HF mice (Supplementary Figures S1–S3), while the underlying mechanism remained to be elucidated. Transmission electron microscopy was used to detect the myocardial ultrastructure to evaluate cardiac injury. As shown in Figure 2A–C, many mitochondria were swollen with abnormal cristae, the mean number of mitochondria were reduced, the ratio of major and minor axes was decreased, and sarcomeres were disordered in the myofibrils in Model group, which was significantly different from mitochondria in Sham group. YQFM treatment improved myocardial ultrastructure and mitochondrial state. Meanwhile, YQFM enhanced Mfn2 expression, as well as reduced Drp1 phosphorylation at Ser616 (Figure 2D,E), which indicated that YQFM could improve the mitochondrial function in HF mice.

### YQFM Ameliorated CAL-Induced Mitochondrial Dysfunction via Regulation of ROS Generation and CaMKII Signaling Pathway in HF Mice

**FIGURE 3:**
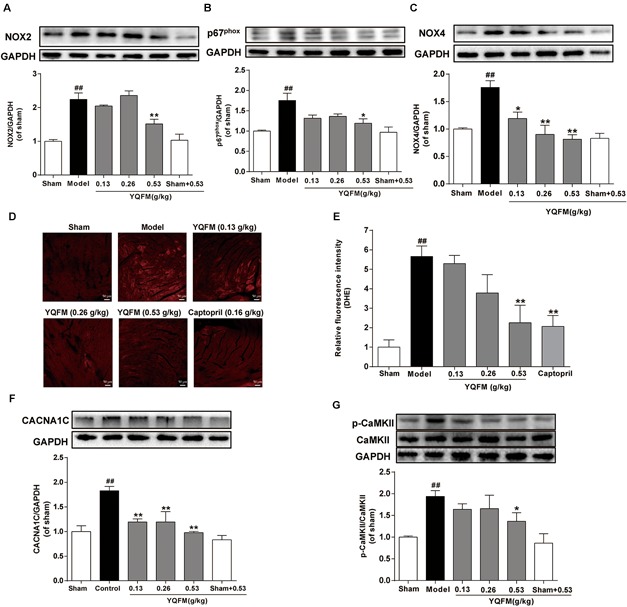
YQFM ameliorated CAL-induced mitochondrial dysfunction via regulation of ROS generation and CaMKII signaling pathways in HF mice. The expression of NOX2 **(A)**, p67^phox^ **(B)**, and NOX4 **(C)** were detected using Western blot analysis. **(D)** Representative images (200 × magnifications) of ROS production in HF myocardium. **(E)** Quantitative analysis of ROS level in heart tissue was calculated. The CACNA1C **(F)**, CaMKII and p-CaMKII **(G)** were detected using Western blot analysis. Results were presented as mean ± SD. ^##^*P* < 0.01 vs. Sham group, ^∗^*P* < 0.05, ^∗∗^*P* < 0.01 vs. Model group. *n* = 3.

To further elucidate the potential mechanism of YQFM, the effects of YQFM on ROS generation have measured. The results revealed that treatment with YQFM markedly reduced the expression of NADPH oxidase subunit, such as NOX2, p67^phox^ and NOX4 (Figure 3A–C). In addition, ROS generation in myocardial tissue was detected by DHE staining, and ROS was reduced by YQFM treatment (Figure 3D,E). Moreover, the CACNA1C expression markedly increased in model group compared with sham group, whereas, YQFM treatment obviously decreased CACNA1C expression (Figure 3F). Further, the phosphorylation of CaMKII markedly increased, while YQFM reduced the expression of phosphorylated CaMKII significantly (Figure 3G). These results indicated that YQFM treatment significantly attenuated CAL-induced ROS generation and activation of CaMKII signaling pathway in HF mice.

### YQFM Ameliorated OGD-Induced Injury and Mitochondrial Dysfunction in NRVMs

**FIGURE 4:**
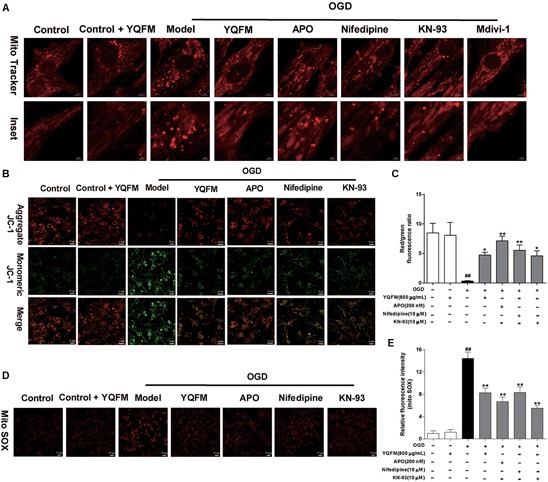
YQFM ameliorated mitochondrial dysfunction in NRVMs subjected to OGD. NRVMs were treated with YQFM (800 μg/mL), APO (200 nM), nifedipine (10 μM) and KN-93 (10 μM), then exposed to OGD 24 h. **(A)** Mitochondrial morphology was analyzed using 63 × oil immersion lens. **(B)** Mitochondrial membrane potential was assessed by probe JC-1. **(C)** Quantitative analysis of mitochondrial membrane potential. **(D)** Mitochondria-derived ROS generation was determined by Mito SOX red fluorescent probe. **(E)** Quantitative analysis of mitochondrial ROS level in NRVMs was calculated. Results were obtained from three independent experiments and were presented as mean ± SD. ^##^*P* < 0.01 vs. Control group, ^∗^*P* < 0.05, ^∗∗^*P* < 0.01 vs. OGD group.

**FIGURE 5:**
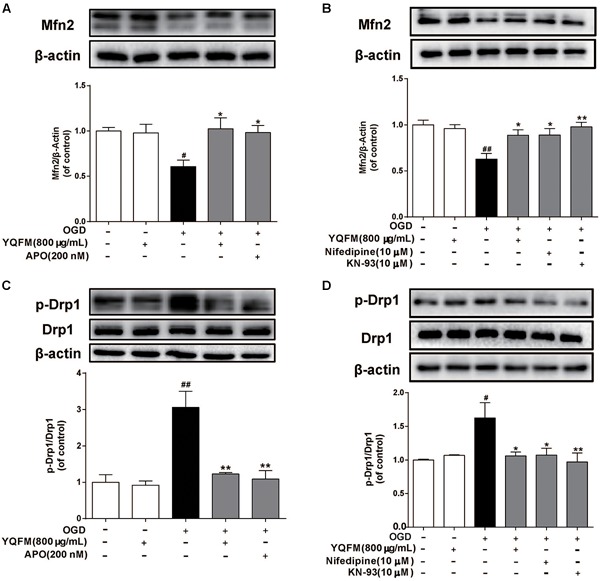
YQFM improved OGD-induced mitochondrial fusion and fission protein expression abnormality. NRVMs were treated with YQFM (800 μg/mL), APO (200 nM), nifedipine (10 μM) and KN-93 (10 μM), then exposed to OGD 24 h. The Mfn2 **(A,B)**, Drp1, p-Drp1 **(C,D)** expression were detected using Western blot analysis. Results were obtained from three independent experiments and were presented as mean ± SD. ^#^P < 0.05, ^##^P < 0.01 vs. Control group, ^∗^*P* < 0.05, ^∗∗^*P* < 0.01 vs. OGD group.

First, the purity of NRVMS was identified as shown in Supplementary Figure S4A. Then, we examined the viability of NRVMs after treatment with YQFM and the result showed YQFM at concentrations of 50–800 μg/mL did not significantly affect cardiomyocytes viability (Supplementary Figure S4B). In addition, exposure of NRVMs (OGD 24 h) led to an obvious decrease in cell viability, nevertheless, YQFM at concentrations of 50–800 μg/mL maintained cell viability significantly (Supplementary Figures S4C,D). Next, as exhibited in Figure 4A, mitochondria became small, round, and punctiform in OGD group, which were different from elongated mitochondria in control group. Besides, Δψm (Figure 4B,C), mitochondrial ROS generation (Figure 4D,E), Mfn2 expression (Figure 5A,B) and Drp1 phosphorylation at Ser616 (Figure 5C,D) between the OGD and control group indicated the damaged mitochondrial function after OGD. After treatment with YQFM, NADPH oxidase inhibitor APO, L-type calcium channels (LTCCs) inhibitor nifedipine and CaMKII inhibitor KN-93, mitochondrial morphology, Δψm and mitochondrial ROS generation were significantly improved, and Mfn2 expression was enhanced, as well as Drp1 phosphorylation at Ser616 was reduced.

### YQFM Protected Against OGD-Induced Mitochondrial Dysfunction Through Inhibiting ROS Generation and CaMKII Signaling Pathways

**FIGURE 6:**
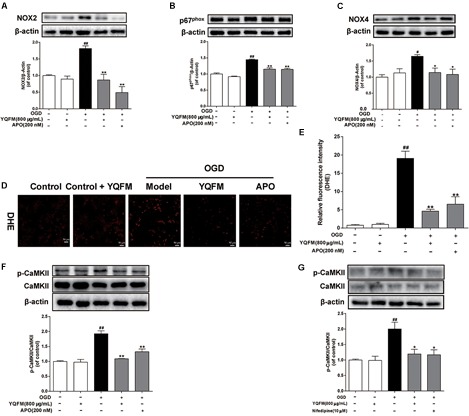
YQFM protected against OGD-induced mitochondrial dysfunction through inhibiting ROS generation and CaMKII signaling pathways. NRVMs were treated with YQFM (800 μg/mL), APO (200 nM) and nifedipine (10 μM), then exposed to OGD 24 h. The NOX2 **(A)**, p67^phox^ **(B)** and NOX4 **(C)** expression were detected using Western blot analysis. **(D)** Representative images (× 200) of ROS production. **(E)** Quantitative analysis of ROS level in NRVMs was calculated. **(F)** The CaMKII and p-CaMKII expression were detected using Western blot analysis. **(G)** The CaMKII and p-CaMKII expression were measured by Western blot analysis. Results were obtained from three independent experiments and were presented as mean ± SD.^##^*P* < 0.01 vs. Control group, ^∗^*P* < 0.05, ^∗∗^*P* < 0.01 vs. OGD group.

As illustrated in Figure 6A–E, YQFM significantly decreased expression of NOX2, p67^phox^, NOX4, and reduced intracellular ROS production, which was similar to the effects of APO. In addition, we found that the phosphorylation of CaMKII significantly enhanced in OGD-induced NRVMs, while YQFM, APO and nifedipine reduced the expression of phosphorylated CaMKII significantly (Figure 6F,G).

## Discussion

Several studies have demonstrated that YQFM was used to treat cardiovascular diseases in clinic (Xu, 2009). However, the underlying mechanisms have yet to be fully elucidated. The present study indicated that YQFM could ameliorate mitochondrial function in HF, partially through inhibiting ROS generation and CaMKII signaling pathways. These findings provide the first evidences that YQFM significantly improved mitochondrial function in CAL-induced HF and OGD-induced NRVMs injury.

As reported previously, the cell changes and apoptosis in signaling molecules are most likely to be associated with mitochondrial dysfunction (Weiss et al., 2003). Myocardial mitochondria are the main energy sources in cardiomyocytes, whose dysfunction promotes pathological development of HF (Elhattab and Scaglia, 2016). Transmission electron microscopy usually used to observe and compare ultrastructure of cells and tissues when higher magnifications than offered by confocal fluorescent microscopy are needed (Tobias et al., 2018). In this study, mitochondrial structure was observed by transmission electron microscopy. It has been found YQFM obviously increase the mean number of mitochondria, reduced ratio of major and minor axes and improved myofibrils disorder in CAL-induced HF mice. In addition, mitochondria constantly undergo fission and fusion, which are controlled by critical regulatory proteins. The cytoplasmic Drp1 is originally located in the cytosol, and when it is activated via phosphorylation of Drp1 at Ser616, it translocates to the foci of future mitochondrial fission sites, leading to fission and subsequent mitochondrial dysfunction (Ong et al., 2010). In addition, Mfn2 is involved in mitochondrial fusion, expression of which is down-regulated in various rat models of cardiac hypertrophy, including spontaneously hypertensive rats, transverse aortic banding, and MI (Fang et al., 2007). As a result, inactivation of Drp1 and activation of Mfn2 contribute to improving mitochondrial dynamic parameter and the delaying development of HF. Our results showed that YQFM not only obviously attenuated mitochondrial function including improving mitochondrial morphology, Δψm and ROS production, also inhibited the phosphorylation of Drp1 and promoted Mfn2 expression in HF mice and OGD-induced NRVMs injury.

Oxidative stress resulting from increased production of ROS or reduced antioxidant defenses, has been implicated in pathophysiology of cardiovascular disease (Hernanz et al., 2014). In addition, mitochondria are central players, both as sources and targets of ROS. Excessive ROS production not only causes nonspecific damage throughout the cell, but also shows to stimulate mitochondrial ROS production and induce mitochondrial dysfunction (Dai et al., 2011). NADPH oxidase, which is involved in the conversion of molecular oxygen to superoxide, is one of the major source for cellular ROS. Major isoforms of NADPH oxidase present in the heart is NOX2 and NOX4 (Sirker et al., 2011). NOX2 is involved in cardiac remodeling after MI, whereas NOX4 is a critical mediator of mitochondrial oxidative stress and mitochondrial dysfunction during HF (Looi et al., 2008; Kuroda et al., 2010). YQFM markedly reduced the expression of NADPH oxidase subunit, such as NOX2, p67^phox^ and NOX4 *in vivo* and *in vitro*. Additionally, both NADPH oxidase inhibitor APO and YQFM decreased intracellular ROS, and improved mitochondrial function. All these findings indicated that YQFM protected against mitochondrial dysfunction through inhibiting ROS generation.

In many cases of cardiac disease, altered Ca^2+^ cycling impels depression of mechanical performance, thus, ameliorating the disorder of Ca^2+^ cycling will be known as an effective therapeutic strategy against HF (Bers, 2000). As we know, antagonists of LTCCs have been applied to treat human cardiovascular diseases for decades. The CaV1.2 channel (pore-forming α1C subunit; referred to herein as CACNA1C) conducts L-type calcium current in cardiomyocytes, where Ca^2+^ enters through the channel and initiates excitation-contraction coupling via Ca^2+^-induced Ca^2+^ release, and reduction of CACNA1C expression refers to LTCCs inhibition (Rothstein and Byron, 2002). In addition to regulating cardiomyocyte contraction, Ca^2+^ influx from LTCCs is also involved in intracellular signaling and gene regulatory events which underlie cardiac disease (Molkentin et al., 1998). For example, multiple studies have suggested that enhanced Ca^2+^ influx via the LTCCs is responsible for cardiac hypertrophy and pathological remodeling of the ventricles (Mukherjee and Spinale, 1998). Moreover, Ca^2+^ entry through LTCCs is quintessential for hypoxic Ca^2+^ overload in the intact heart, and provided evidence of hypoxia-induced cardiac Ca^2+^ overload and its relation to mitochondrial dysfunction and membrane injury (Zhang et al., 2013). And as a major active component of YQFM, ginsenoside Rb1 has been reported to inhibit L-type calcium current significantly, also suppress the opening of LTCCs in ischemic cardiomyocytes (Zhang et al., 2007; Pei et al., 2011). Meanwhile, ginsenoside Rd could inhibit L-type calcium current in rat ventricular myocytes (Lu et al., 2015). In current study, we found that YQFM treatment obviously decreased CACNA1C expression in CAL-induced HF mice. YQFM and LTCCs inhibitor, nifedipine also improved mitochondrial function in OGD-induced NRVMs. These results suggested that hypoxia induced LTCCs impaired in NRVMS and YQFM possibly inhibited this process.

Additionally, CaMKII is phosphorylated at an autophosphorylation site (Thr 286/287) in the regulatory domain once Ca^2+^/CaM elevations occur at high frequency or for a long time (De Koninck and Schulman, 1998). Activation of CaMKII in failing hearts also promotes calcium leak and causes mitochondrial damage (Zhang et al., 2003). Moreover, CaMKII inhibition protects against HF and cardiomyocyte death in response to MI (Zhang et al., 2005). In present study, CaMKII inhibitor KN-93 could ameliorate mitochondrial function. This phenomenon showed that CaMKII activation damaged mitochondrial function in OGD-induced NRVMs. Furthermore, YQFM, APO, and LTCCs inhibitor, nifedipine could down-regulate expression of p-CaMKII and improve mitochondrial function *in vitro*. Although ROS can oxidize CaMKII in a manner of ox-CaMKII (Erickson et al., 2008), numerous previous reports have shown that ROS increases the Ca^2+^ influx through LTCCs (Bagchi et al., 1997) and activity of the Na^+^/H^+^ exchanger (Gen et al., 2001), thereby lead to an increase in cytosolic Ca^2+^ and phosphorylation of CaMKII. So it’s possible that YQFM inhibited CaMKII activation through reducing ROS production and inhibiting LTCCs. In present research, there are some deficiencies, such as YQFM’s effects on links of ROS, intracellular Ca^2+^ and CaMKII, L-type calcium channel current and calcium transients. Further studies are required to explain the problem more fully, and the future use of the whole-cell patch clamp technique and dual excitation fluorescence photomultiplier system to record L-type calcium current and calcium transients could be relevant strategies (Fares et al., 2010; Ceylan-Isik et al., 2011).

In summary, our results indicated that YQFM ameliorated mitochondrial function in HF, partially through inhibiting ROS generation and CaMKII signaling pathways. All of these findings provided another pathway of YQFM, in terms of gaining more insight on the mechanism of YQFM in clinical treatment of HF.

## Ethics Statement

All procedures were operated in accordance with National Institutes of Health Guide for the Care and Use of Laboratory Animals, and the protocols used were also consistent with the Animal Ethics Committee of China Pharmaceutical University, China Pharmaceutical University, Nanjing, China.

## Author Contributions

YuZ, FL, and JK designed the research. YuZ, LZ, and XF produced animal model and measured the samples. FL and WY conducted the cell experiments. YaZ, XF, and FL analyzed data. YuZ wrote the manuscript. JK and BY provided technical and material support. FL, JK, and BY critically revised the manuscript.

## Conflict of Interest Statement

The authors declare that the research was conducted in the absence of any commercial or financial relationships that could be construed as a potential conflict of interest.
