# Supplementary material for: YiQiFuMai Powder Injection Attenuates Coronary Artery Ligation-Induced Heart Failure Through Improving Mitochondrial Function via Regulating ROS Generation and CaMKII Signaling Pathways
Source: Front Pharmacol. 2019 Apr 10;10:381. doi: 10.3389/fphar.2019.00381 (PMC6470332; doi:10.3389/fphar.2019.00381)
Supplement: Supplementary file 1 [file Data_Sheet_1.docx]

**Supplementary materials**

**
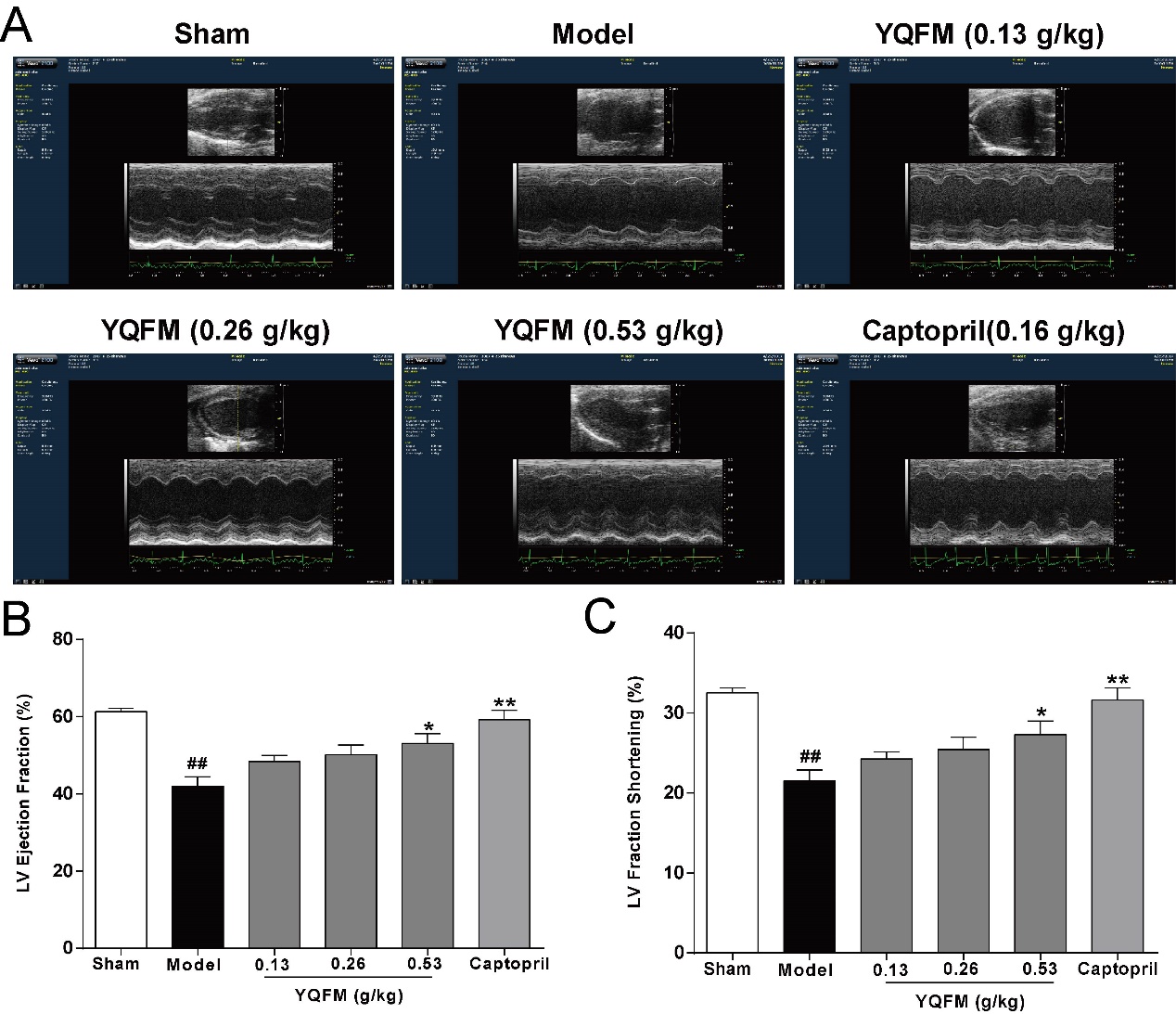
**

**Figure S1** YQFM improved the cardiac systolic function in CAL-induced HF mice. (A) Representative images of M-mode echocardiograms. Effect of YQFM on LV Ejection Fraction (B), LV Fraction Shortening (C) changes after 14 days of CAL in mice. Results were presented as mean ± SD. ^##^P < 0.01 vs. Sham group, ^*^P <0.05, ^**^P < 0.01 vs. Model group. n=8.


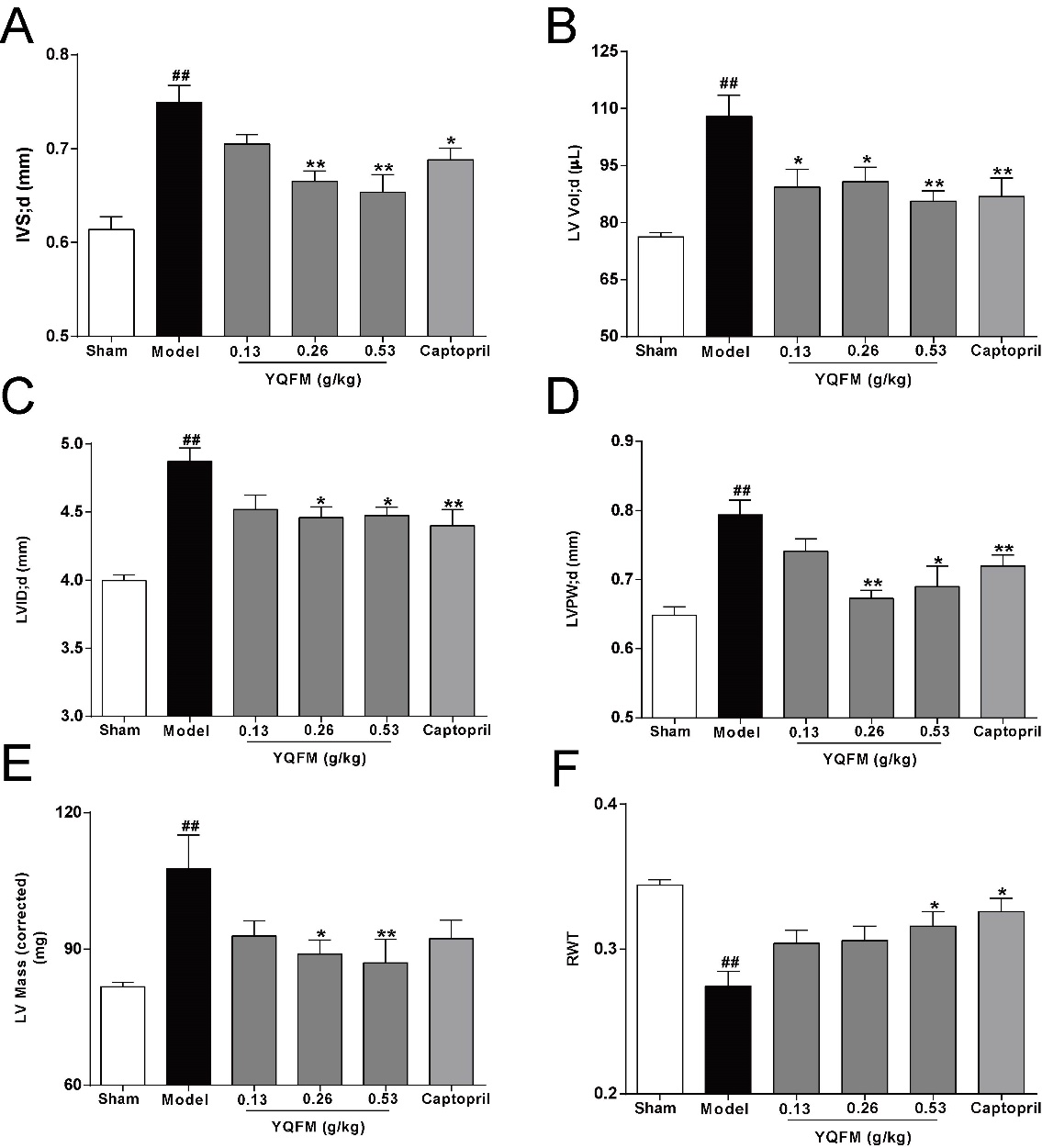


**Figure S2** YQFM inhibited the cardiac hypertrophy in CAL-induced HF mice. These parameters were calculated: (A) IVS;d, (B) LV Vol;d, (C) LVID;d, (D) LVPW;d, (E) LV Mass (corrected) and (F) RWT. Results were presented as mean ± SD. ^##^P < 0.01 vs. Sham group, ^*^P <0.05, ^**^P < 0.01 vs. Model group. n=8.


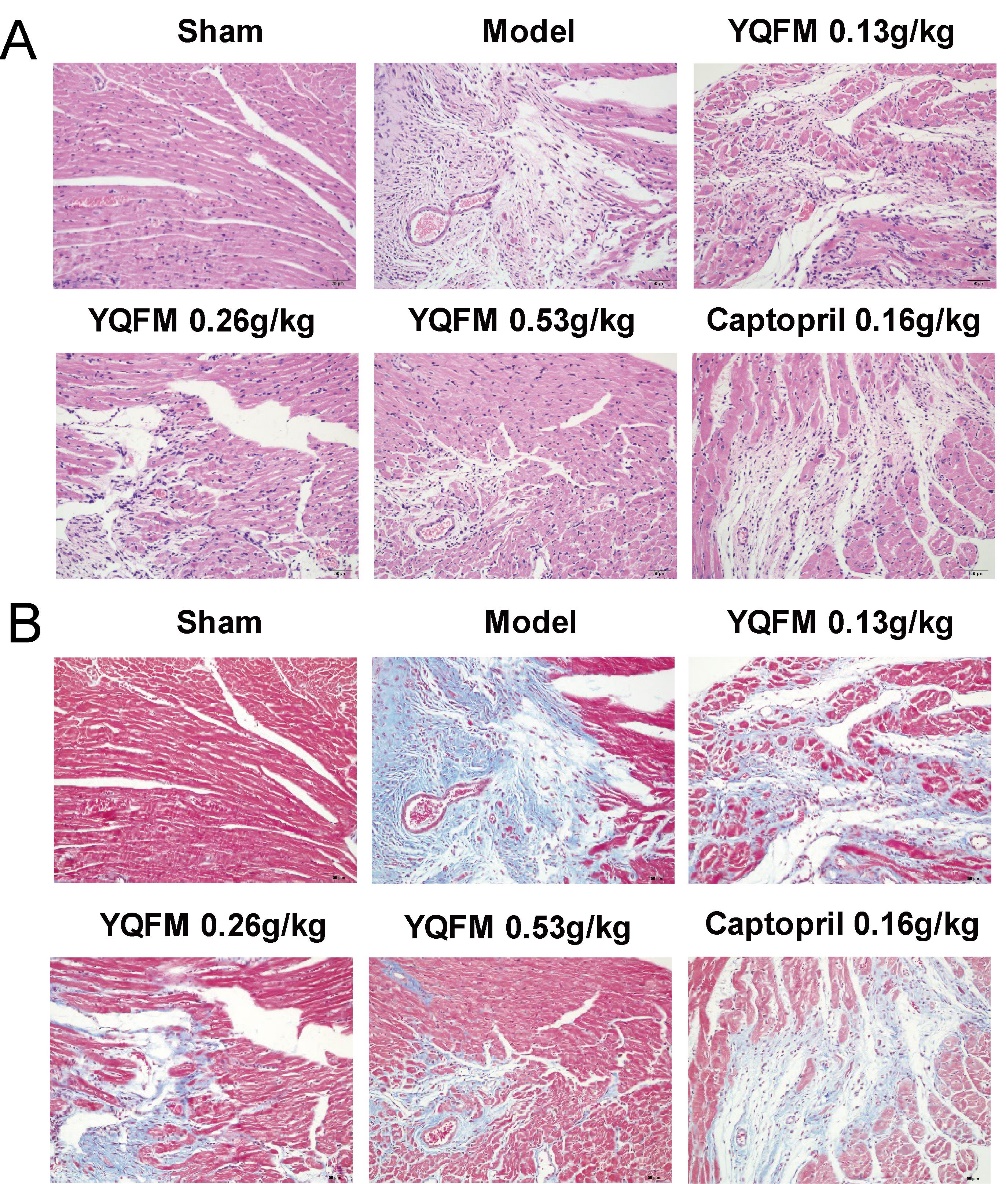


**Figure S3** YQFM ameliorated cardiac pathological changes and fibrosis in CAL-induced HF mice. (A) Histopathological changes of representative heart sections were measured by hematoxylin-eosin staining (×200). (B) Myocardial fibrosis was measured by Masson’s trichrome staining (×200). n=6.

**
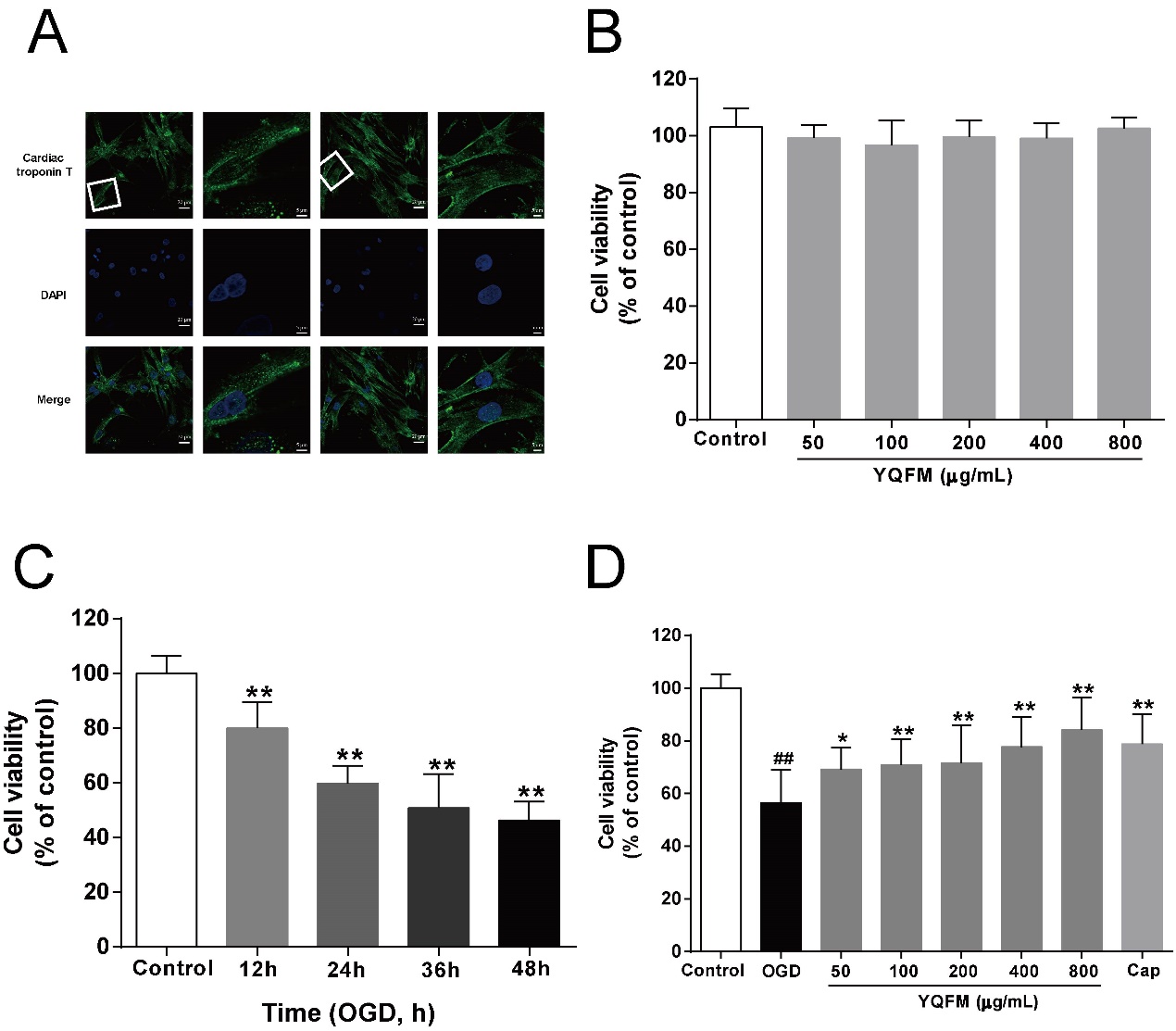
**

**Figure S4** YQFM decreased OGD-induced NRVMs injury. (A) Identification of NRVMs. NRVMs were marked by anti-cTnT antibody and analyzed using 63×oil immersion lens. (B) NRVMs were treated with YQFM at the concentration of 25, 50, 100, 200, 400, 800 μg/mL for 24 h. (C) Time course of the viability of NRVMs induced by OGD. Cell viability was measured by the MTT assay described in method after 12, 24, 36, 48 h OGD. Results were obtained from three independent experiments and were presented as mean ± SD. ^**^P < 0.01 vs. Control group. (D) NRVMs were treated with YQFM (25-800 μg/mL) and then exposed to OGD for 24 h. The cell viability was measured by MTT assay described in method. Cap = Captopril (positive control). Results were obtained from three independent experiments and were presented as mean ± SD. ^##^P < 0.01 vs. Control group, ^*^P <0.05, ^**^P < 0.01 vs. OGD group.
